# Continuing Fragmentation of a Widespread Species by Geographical Barriers as Initial Step in a Land Snail Radiation on Crete

**DOI:** 10.1371/journal.pone.0062569

**Published:** 2013-05-01

**Authors:** Jan Sauer, Jens Oldeland, Bernhard Hausdorf

**Affiliations:** 1 Department of Chemical Ecology, University of Bielefeld, Bielefeld, Germany; 2 Zoological Museum, University of Hamburg, Hamburg, Germany; 3 Biodiversity, Evolution and Ecology of Plants, Biozentrum Klein Flottbek, University of Hamburg, Hamburg, Germany; University of Arkansas, United States of America

## Abstract

The phylogeographic structure of the land snail *Xerocrassa mesostena* on Crete inferred from AFLP markers and mitochondrial *cox1* sequences can be explained by three mechanisms: gene flow restriction, population expansion and leptokurtic dispersal. Gene flow restriction by geographic barriers caused subdivision of the gene pool into distinct clusters. Population expansion was probably facilitated by deforestation of Crete in the postglacial. Newly available areas were colonized by leptokurtic dispersal, i.e. slow active expansion resulting in isolation by distance within the clusters and occasional long distance dispersal events that resulted in departures from the isolation by distance model. Less than one percent of the AFLP markers show correlations with environmental variables. Random phylogeographic breaks in the distribution of the mitochondrial haplotype groups indicate that single locus markers, especially mitochondrial DNA, might result in a misleading picture of the phylogeographic structure of a species. Restriction of gene flow between metapopulations caused by geographical barriers can interact with sexual selection resulting in the differentiation of these metapopulations into separate species without noticeable ecological differentiation. Evidence for gene flow between parapatrically distributed evolutionary units representing different stages of the speciation process suggests that the ongoing process of fragmentation of the *X. mesostena* complex might be an example for parapatric speciation. The lack of ecological differentiation between these units confirms theoretical predictions that divergent selection for local adaptation is not required for rapid speciation.

## Introduction

Geographical isolation, selection and genetic drift may result in geographic differentiation of populations and finally in speciation. The relative importance of these processes and the conditions which determine which of these processes becomes the pacemaker of speciation are still unclear [1]–[2]. Cases of ongoing speciation need to be studied in order to understand the role of these processes in speciation.

Snails often show distinct patterns of morphological and genetic differentiation of metapopulations and are classical study objects for investigating the roles of history and selection in the origin of diversity [3]. They provide paradigms for population structure due to selection as well as population structure resulting from historical or current geographical barriers. An example of population structure resulting from selection without geographical isolation is the periwinkle *Littorina saxatilis*. In response to selection by crabs in the lower littoral zone and to wave action in the upper littoral zone, differently adapted clusters characterized by different shell forms evolved convergently along several European coasts [4]–[6]. On the contrary, some studies of genetic differentiation in land snails revealed no evidence for selection. Thus, it was inferred that ‘area effects’ in *Cepaea nemoralis* [7], genetic differentiation in *Partula taeniata* [8], and the differentiation of subspecies in *Charpentieria itala* [9] resulted from historical separation of metapopulations.

The radiation of the helicoid land snail genus *Xerocrassa* on Crete has resulted in ten endemic species [10]. All these species are xerophilic and live in open, dry habitats, which are currently available almost throughout Crete. This radiation was mainly non-adaptive and has been triggered by sexual selection [11]. *X. mesostena* is the most widespread of these species, and is paraphyletic with respect to several of the other endemic *Xerocrassa* species in a mitochondrial gene tree [11]. Most other species or species complexes probably originated from *X. mesostena* by peripatric speciation [12]. There are deep splits in *X. mesostena* [12]–[13] that indicate that the speciation process is continuing. Thus, we can study metapopulations in an early stage of differentiation within *X. mesostena* that may evolve into separate species.

We investigated the importance of geographical isolation and selection for the geographic differentiation of *X. mesostena* and their role as initial factors triggering speciation in the Cretan *Xerocrassa* radiation. We studied the population structure of *X. mesostena* using amplified fragment length polymorphism (AFLP) markers [14] and compared it with the phylogeographic pattern revealed by mitochondrial sequences to infer the importance of geographic barriers for the differentiation process. Furthermore, we applied a landscape genetic approach based on AFLP data to investigate the role of natural selection by environmental variables in the differentiation process [15].

## Materials and Methods

### Sampling

*Xerocrassa* specimens were sampled in July/August and September/October 2004 and September/October 2005. These snail species are categorized as Least Concern in the Red List of the International Union for Conservation of Nature (IUCN), and thus no specific permission was required for this study. Mitochondrial *cox1* sequences and AFLP data were determined from 95 specimens from 84 localities across Crete covering all known morphotypes. The locality, voucher and AFLP data for the *Xerocrassa* specimens used in this study can be found in Table S1.

### DNA extraction, amplification and sequencing

Total genomic DNA was extracted from snail foot tissue samples preserved in 100% isopropanol following the protocol proposed by Sokolov [16] as modified by Sauer and Hausdorf [11]. Fragments of the mitochondrial cytochrome c oxidase subunit I (*cox1*) gene were amplified using Polymerase Chain Reaction (PCR) and sequenced as described by Sauer and Hausdorf [11].

### Sequence analysis

Forward and reverse sequences were assembled using ChromasPro version 1.33 (Technelysium). The sequences were aligned with the CLUSTAL W algorithm [17] as implemented in MEGA version 4.0 [18] with the default settings. The sequences generated for this study have been deposited in GenBank under the accession numbers KC710247-KC710294. The accession numbers for all individuals analysed in this study are listed in Table S1. The used alignment is available at TreeBASE (http://www.treebase.org, accession number S13717).

### AFLP

The AFLP data were generated as described by Sauer and Hausdorf [13] and can be found in Table S1.

### Phylogenetic analyses

Models of sequence evolution for the distance calculations and the maximum-likelihood analysis based on the *cox1* sequences were chosen using ModelTest version 3.7 [19] based on the Akaike Information Criterion (AIC). The maximum-likelihood analysis was conducted with Treefinder [20]–[21]. Individuals of *Xerocrassa subvariegata* and *X. grabusana* were used as outgroups for the phylogenetic reconstruction. Confidence values for the edges of the maximum-likelihood tree were computed by bootstrapping (100 replications; [22]).

### Tests for neutral evolution of the mtDNA and for range expansions

We tested whether the *cox1* sequences were evolving neutrally with the McDonald and Kreitman [23] test. To infer population size changes from the haplotype data, we used Fu's *Fs* statistic [24]. Negative departures of this statistic from zero indicate an excess of low-frequency alleles expected in the course of a population expansion [24]. Confidence intervals of *Fs* were determined by coalescent simulations. The calculations were performed with DnaSP version 4.10.9 [25].

### Inferring phylogeographic structure based on AFLP data

We used the Bayesian program STRUCTURE [26]–[27] to investigate the phylogeographic structure of *Xerocrassa mesostena* based on the AFLP data without a priori grouping of individuals into populations. We explored the pattern of admixture by varying the number of groups *K* in the dataset and assigning proportions of each individual to these groupings using STRUCTURE version 2.3.1 with the model with admixture. Following Evanno et al. [28] 20 runs with 10,000 iterations after a burn-in of 10,000 iterations were carried out for each cluster number *K* from 2 to 15. We used the mean estimates of the posterior probabilities of the data for a given cluster number *K* (*L*(*K*)) and the statistic Δ*K*  =  m(|*L*(*K*+1)-2*L*(*K*)+*L*(*K*-1)|)/*s*[*L*(*K*)] proposed by Evanno et al. [28] to estimate the number of clusters *K*. For the *K* determined in that way, a run with a burn-in of 30,000 iterations followed by 100,000 iterations was performed to infer the admixture.

As alternative to STRUCTURE, we investigated the phylogeographic structure based on the AFLP data using Gaussian clustering [29] as implemented in the program package PRABCLUS version 2.1–1 [30], an add-on package for R [31]. This non-hierarchical clustering method does not depend on the assumption of Hardy-Weinberg equilibrium within populations and provides a decision about the number of meaningful clusters. Gaussian clustering operates on a dataset where the cases are defined by variables of metric scale. Therefore, we performed a non-metric multidimensional scaling [32] based on Jaccard distances derived from the AFLP data.

Furthermore, we constructed a neighbour-net with SplitsTree4 version 10 [33] using Jaccard distances calculated with PhyloTools version 1.32 [34] based on the AFLP data to visualize the relationships between the investigated *X. mesostena* individuals.

### Concordance of genetic clusters with geography

The geographical separation of the AFLP based clusters and *cox1* haplotype groups was assessed using one-way analysis of similarities (ANOSIM) as implemented in PAST v.1.95 [35]. ANOSIM is a non-parametric test of differences between two or more groups that is based on comparing distances between groups with distances within groups [36]. Any distance measure may be used. To quantify the geographical separation of the genetic clusters, Euclidean distances were calculated based on the geographic coordinates of the localities of the examined specimens. Large positive values of the test statistic *R* (up to 1) signify dissimilarity between groups. The significance was computed by 10,000 permutations of group membership. The *p* values were Bonferroni corrected for multiple comparisons.

### Identifying barriers to gene flow and long distance dispersal events

Barriers to gene flow are typically indicated by high genetic distances across short geographical distances. We calculated geographic distances with ArcGIS v.9.3 [37] and used Jaccard distances for the AFLP data and GTR+G distances calculated with PAUP*4.0 beta 10 [38] for the *cox1* sequences. We determined the pairs of individuals that belong to both, the quartile of the highest genetic distances and the quartile of the lowest geographic distances. Barriers to gene flow were visualized by plotting lines between these specimen pairs on a map.

Long distance dispersal events will result in low genetic distances across large geographical distances. Thus, we determined the pairs of individuals that belong to both, the quartile of the lowest genetic distances and the quartile of the highest geographic distances and visualized potential long distance dispersal events by plotting lines between these specimen pairs on a map.

### Isolation by distance and subdivision into clusters

We used distance-based redundancy analysis (dbRDA, [39], [40]) as implemented in DISTLM [41] to investigate whether the phylogeographic structure of *X. mesostena* can be better described as isolation by distance [42] or as subdivision into clusters. We performed a multivariate multiple regression analysis of the genetic distance matrices (Jaccard distances based on AFLP data respectively GTR+G distances based on *cox1* sequences) and latitude and longitude as spatial variables to infer how much of the genetic variability can be explained by isolation by distance. *p* values were estimated from 9,999 permutations of the spatial variables. Then a set of *n*-1 dummy variables (*n*  =  number of AFLP based clusters or *cox1* haplotype clusters) indicating the genetic cluster affiliation of each individual (a 0/1 table) was constructed. The cluster affiliation of the individuals belonging to the last (*n*th) cluster would be redundant information. The genetic distance matrices were analysed using dbRDA with the set of variables indicating the genetic cluster affiliation of each individual as predictor variable set and with the spatial variables set as covariates to determine the importance of the subdivision into clusters. We used 9,999 permutations of the cluster affiliations to estimate *p* values. Additionally, the within cluster variation was analysed separately for the clusters either based on the AFLP data or the *cox1* sequences.

### Association between genetic data and environmental variables

In order to identify selection pressures potentially influencing the population genetic structure and AFLP loci that are linked to adaptations to these selection pressures, we used the spatial analysis method implemented in the program MatSAM [15]. The program performs univariate logistic regressions to test for association between presence/absence of an allele and an environmental variable. The significance of the logistic regression model for each locus is assessed with likelihood ratio (*G*) as well as with Wald tests [15]. Possible selection on a locus is inferred only if both tests were significant after Bonferroni correction for multiple testing. We downloaded eight monthly gridded environmental parameters (precipitation (sum); precipitation (coefficient of variation); number of wet-days; relative humidity; mean temperature; mean diurnal temperature range; sunshine; windspeed) from the websites of the Climatic Research Unit (http://www.cru.uea.ac.uk). The data are divided in raster cells with a resolution of 10 minutes (34 raster cells on Crete) that were derived from interpolations based on a data set of weather station means for the period from 1961 to 1990 [43]. For each locality where *Xerocrassa mesostena* individuals were sampled the environmental parameters were extracted. In addition to the monthly values (n = 105), we calculated for each parameter either annual sums or averages. Four individuals had to be excluded because their sampling localities could not be assigned to a raster cell. All processing was done using SAGA-GIS version 2.0.8 [44]. The data used for the MatSAM analysis are compiled in Table S2.

## Results

### Network and phylogeographic structure based on AFLP data

**Figure 1:**
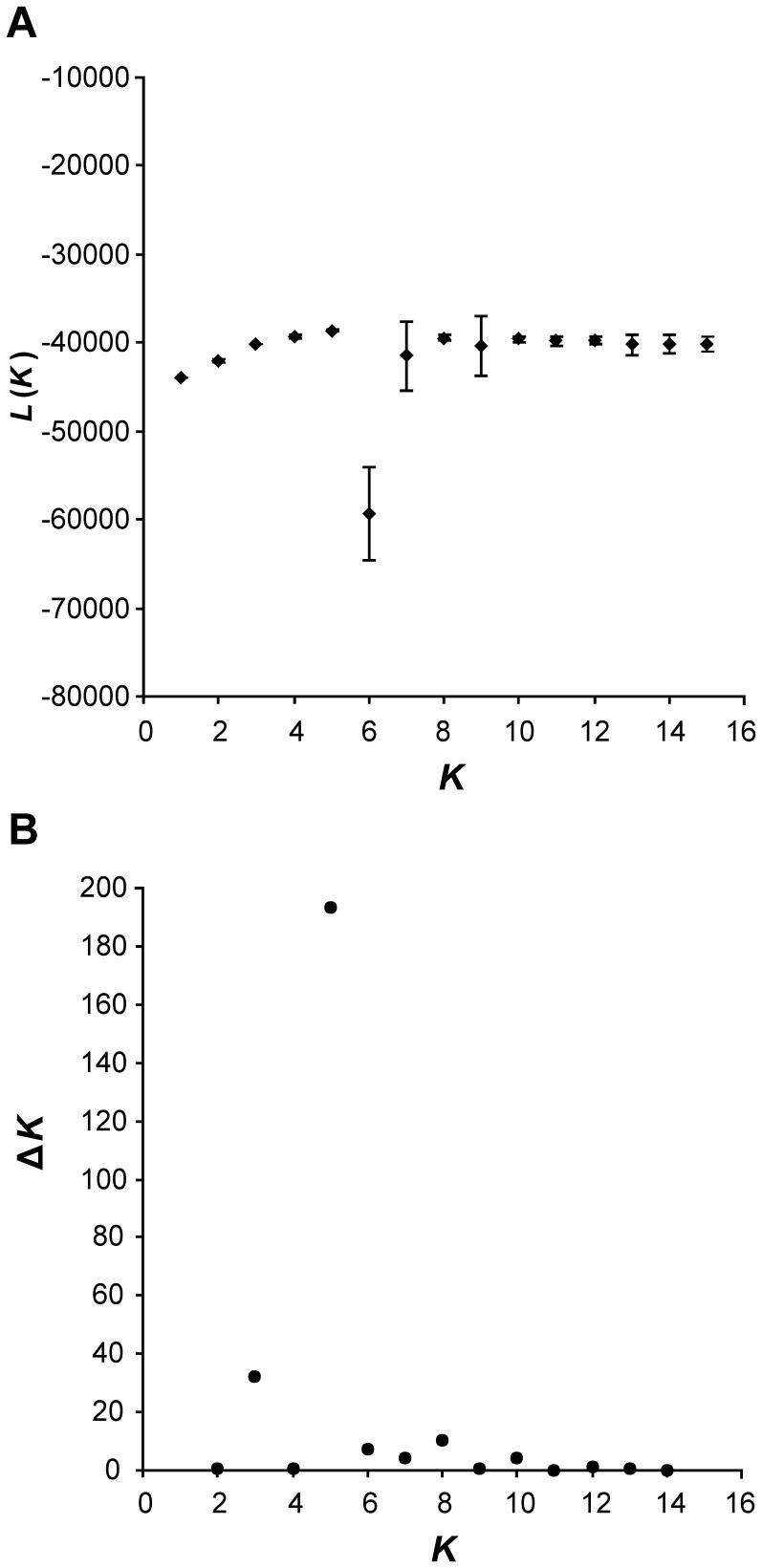
Results of the STRUCTURE analysis of the AFLP data of *Xerocrassa mesostena* for different cluster numbers *K* from 2 to 15. A. Mean estimates of the posterior probabilities of the data for a given *K* (±SD). B. Δ*K* (following Evanno et al. [28]).

**Figure 2:**
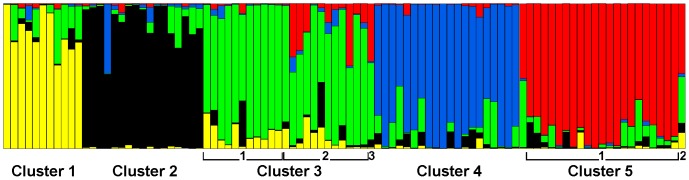
Results of the admixture analysis with the AFLP data of *Xerocrassa mesostena* with STRUCTURE for *K* = 5. Individuals are sorted into clusters and the clusters are ordered from west to east. The division of clusters 3 and 5 as found in the neighbor-net network (Fig. 3) is also indicated.

**Figure 3:**
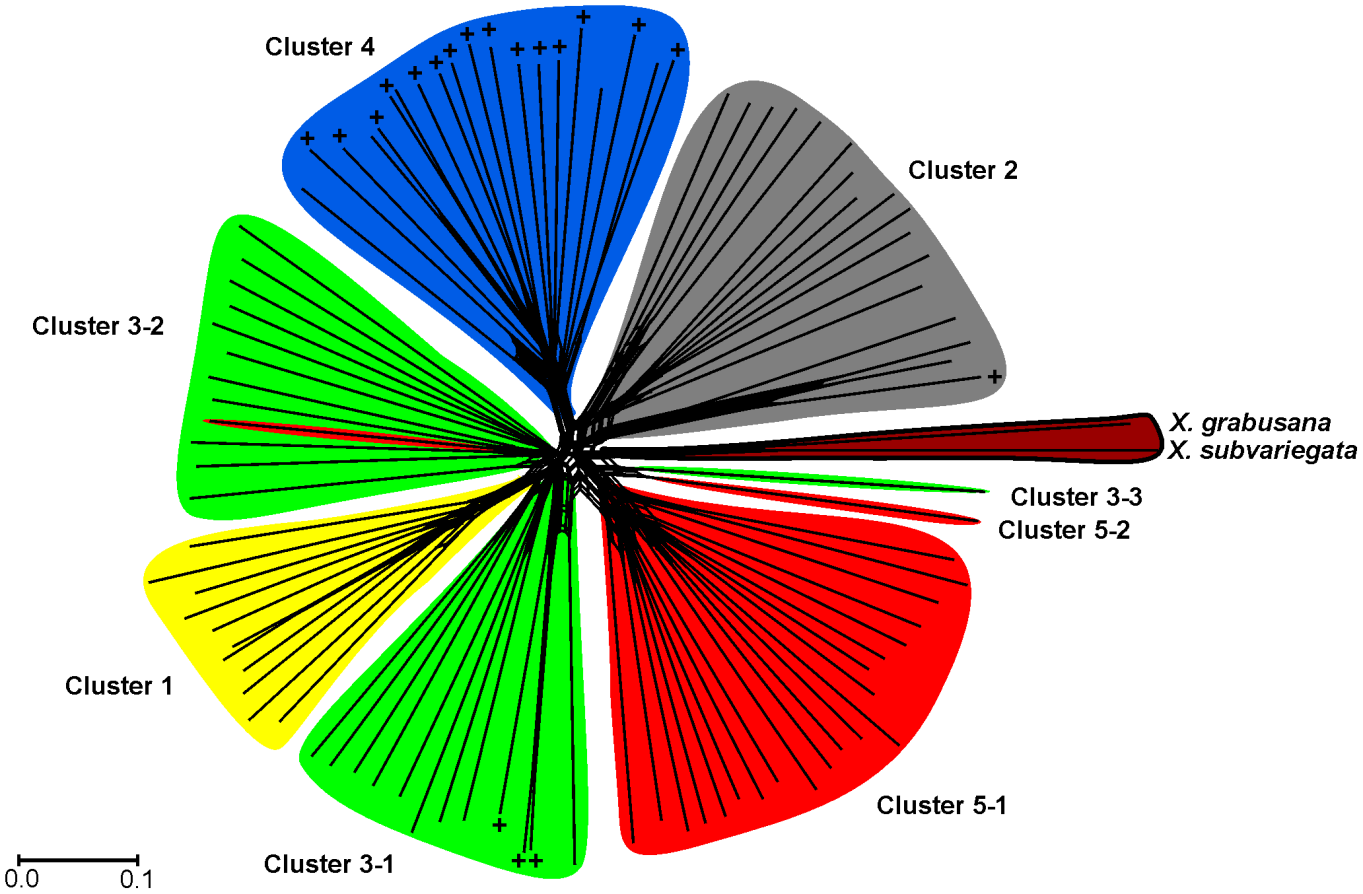
Neighbor-net network based on Jaccard distances between AFLP data of 95 *Xerocrassa mesostena* individuals and *X. subvariegata* and *X. grabusana* as outgroups. The clusters delimited using STRUCTURE are shown for comparison. The *X. mesostena* individuals that are characterized by mitochondrial haplotypes of the ‘Psiloritis’ group are indicated by +.

1328 AFLP fragments of 70–322 bases length resulting from six primer combinations were scored in 95 individuals of *X. mesostena* and one individual of *X. subvariegata* and of *X. grabusana* as outgroups (Table S1). Both the mean estimates of the posterior probabilities of the data calculated in admixture analyses with STRUCTURE (Fig. 1A) and the statistic *ΔK* proposed by Evanno et al. [28] to estimate the number of clusters *K* (Fig. 1B) showed a distinct maximum for *K* = 5 indicating that our samples of *X. mesostena* can be classified into five clusters based on the AFLP data. As expected, individuals showed admixture, especially with the neighbouring clusters (Fig. 2). The five STRUCTURE clusters were in good accordance with the main groupings in a neighbor-net based on the AFLP data (Fig. 3). Only a few individuals with high degrees of admixture according to the STRUCTURE analysis were placed in different or separate groups. Cluster 3 is subdivided into a northern and a southern part and the single individual isolated in eastern Lasithi.

**Figure 4:**
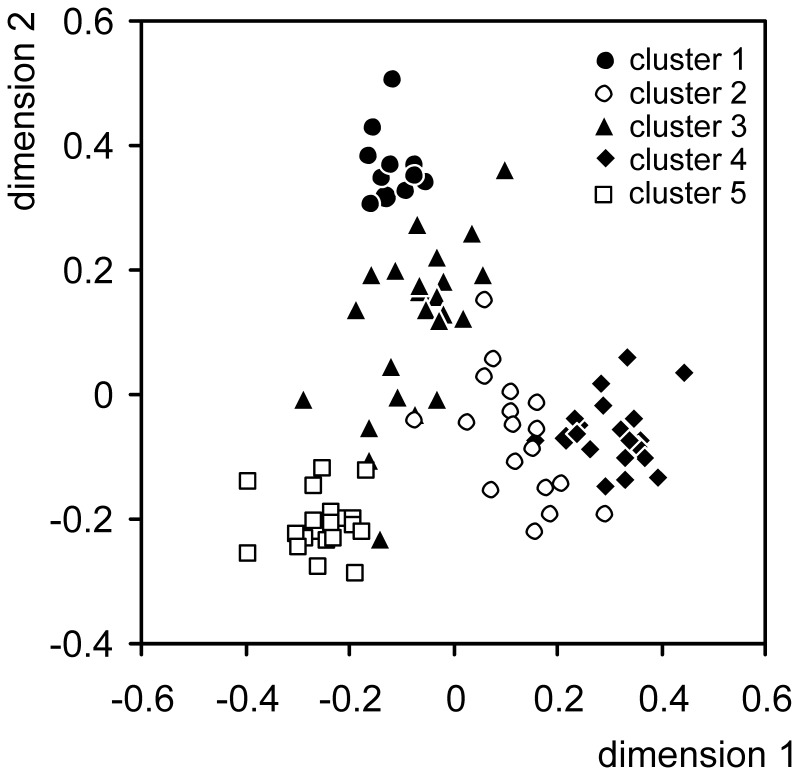
First two dimension of a non-metric multidimensional scaling based on Jaccard distances between the AFLP data of the *Xerocrassa mesostena* individuals. Clusters delimited with Gaussian clustering are shown.

The Gaussian clustering based on the AFLP data of the 95 *X. mesostena* individuals resulted also in five clusters (Fig. 4). The clustering was almost identical with that found in the STRUCTURE analysis. Only three individuals from areas where clusters abut were assigned into different clusters. These three individuals had high degrees of admixture according to the STRUCTURE analysis. The second largest admixture component was always from the cluster occurring in the adjacent region to which Gaussian clustering assigned the particular individual.

**Figure 5:**
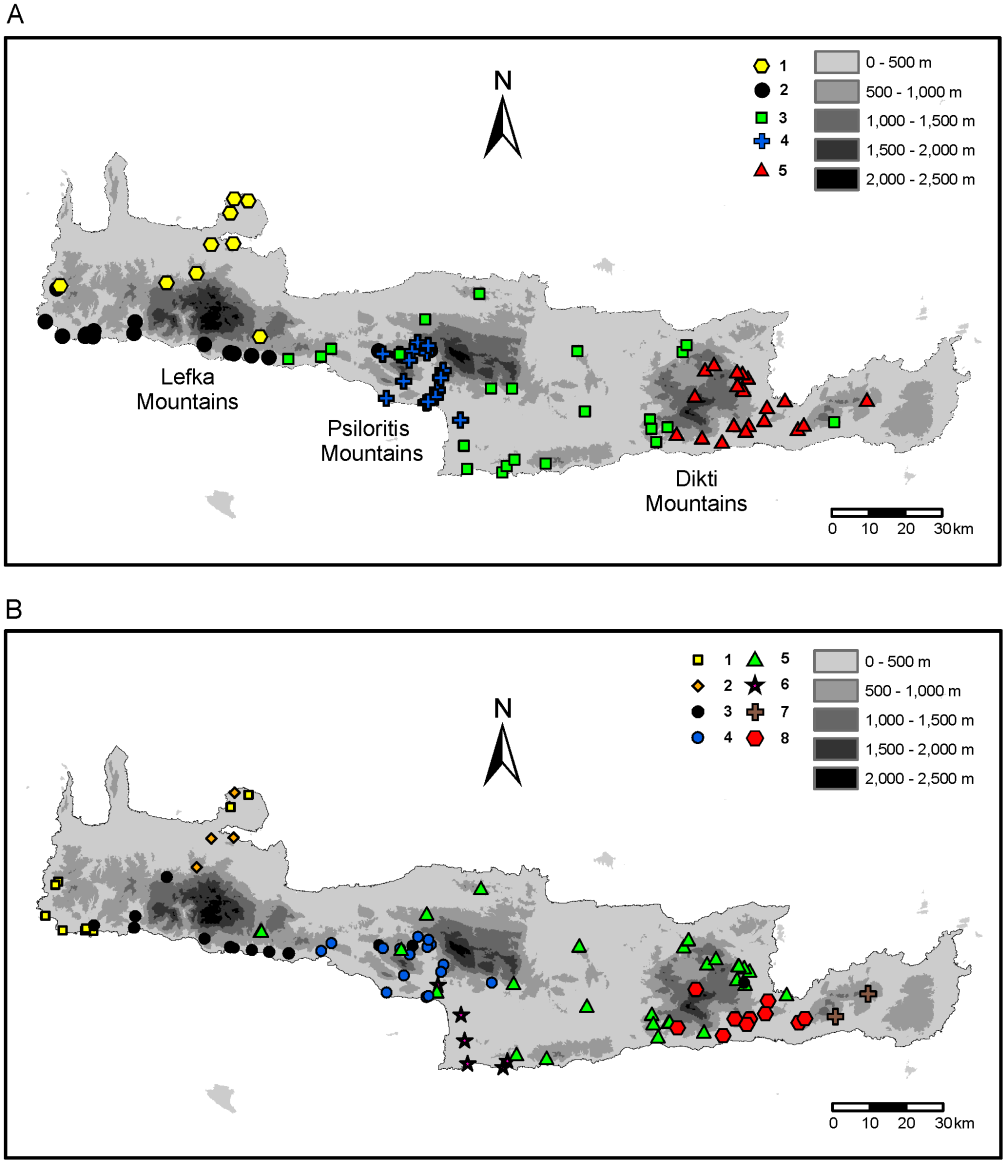
Geographic distribution of the genetic clusters within *Xerocrassa mesostena* on Crete. A. Distribution of the AFLP based clusters delimited using STRUCTURE. B. Distribution of the mitochondrial haplotype groups.

The ANOSIM results showed that the clusters identified by STRUCTURE based on the AFLP data were geographically well separated (global *R* = 0.7568, *p*<0.0001). The pairwise comparisons confirmed that all five clusters were significantly separated (Table 1). The geographical boundaries of the clusters corresponded generally with current barriers, namely the Lefka, Psiloritis and Dikti Mountains as well as the Messara Plain (Fig. 5A). The first of the five STRUCTURE clusters was distributed north of the Lefka Mountains to the Akrotiri Peninsula. The second cluster was found south of the Lefka Mountains along the coast and extended into the region southwest of the Psiloritis Mountains. A widespread cluster occupied the region from the Lefka Mountains to the west side of the Dikti Mountains. This cluster was divided in the neighbour-net (Fig. 3) into two groups. The one group (cluster 3–1) included the specimens from southern-central Crete eastwards to the southwestern slope of the Dikti Mountains as well as one individual from the region north of the Psiloritis Mountains and one individual from the region east of the Dikti Mountains. The other group (cluster 3–2) included the specimens from the region between the Lefka and the Psiloritis Mountains eastwards to the north-western slope of the Dikti Mountains. Finally, there were two more restricted clusters. One is bordered in the north and east by the Psiloritis Mountains and in the southwest by the Messara plain. The other was found from the Dikti Mountains eastwards.

**Table 1:** Pairwise *R* values calculated using an ANOSIM of the AFLP based clusters delimited using STRUCTURE.

	Cluster 1	Cluster 2	Cluster 3	Cluster 4	Cluster 5
Cluster 1		0.311**	0.760***	0.988***	1.000***
Cluster 2	0.311**		0.729***	0.781***	0.996***
Cluster 3	0.760***	0.729***		0.335***	0.532***
Cluster 4	0.988***	0.781***	0.335***		1.000***
Cluster 5	1.000***	0.996***	0.532***	1.000***	

Significances were obtained by 10,000 permutations and are indicated by asterisks (** < 0.01 and *** < 0.001). The *p* values were Bonferroni corrected for multiple comparisons.

### Mitochondrial gene tree and phylogeographic structure based on *cox1* sequences

**Figure 6:**
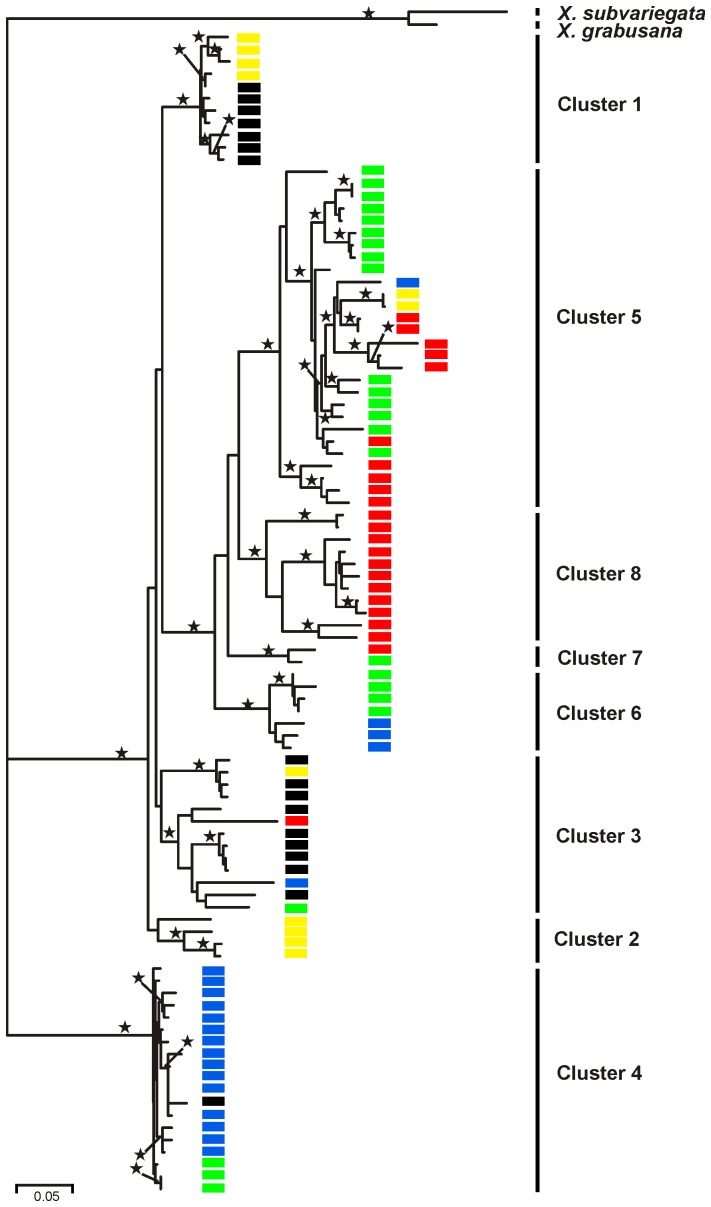
Maximum-likelihood tree based on partial *cox1* sequences of 95 *Xerocrassa mesostena* individuals and *X. subvariegata* and *X. grabusana* as outgroups. Haplotype clades are labelled to the right of the tree. Bootstrap support values larger than 75% are indicated by asterisks above the branches. For each individual the AFLP cluster as delimited using STRUCTURE is indicated by the same colour as in Figure 2.

Separate models for the three codon positions of the *cox1* sequences as determined by ModelTest were used for the maximum-likelihood analysis (1. codon positions: TN+G, 2. codon positions HKY+I, 3. codon positions GTR+I+G), because the resulting tree had a lower AIC value than the tree based on a uniform model for the complete dataset (634 bp). Based on the maximum-likelihood tree (Fig. 6), the *cox1* haplotypes of the 95 *Xerocrassa mesostena* individuals can be classified into eight major groups. The deepest split separated haplotypes from individuals inhabiting a region southwest of the Psiloritis Mountains (clade 4) from all other haplotypes of *X. mesostena*. The ‘Psiloritis’ group is so deeply separated from the rest of the *X. mesostena* haplotypes (average GTR+G distance of ‘Psiloritis’ haplotypes to other *X. mesostena* haplotypes was 18.8%) that it might be taken for a cryptic species, if only mitochondrial sequences were available. The *cox1* haplotype groups are restricted to subregions of Crete, but several of them have overlapping ranges (Fig. 5B). The ANOSIM confirmed that these haplotype groups are generally geographically distinct (global *R* = 0. 5928, *p*<0.0001), but that not all eight groups can be geographically separated (Table 2).

**Table 2:** Pairwise *R* values calculated using an ANOSIM of the *cox1* haplotype groups.

	Cluster 1	Cluster 2	Cluster 3	Cluster 4	Cluster 5	Cluster 6	Cluster 7	Cluster 8
Cluster 1		0.160	0.268	0.989***	0.885***	1.000***	1.000	1.000***
Cluster 2	0.160		0.151	0.998	0.789***	1.000	1.000	1.000*
Cluster 3	0.268	0.151		0.633***	0.598***	0.590**	0.849	0.816***
Cluster 4	0.989***	0.998**	0.633***		0.478***	0.467*	1.000	1.000***
Cluster 5	0.885***	0.789***	0.598***	0.478***		0.242	0.323	0.004
Cluster 6	1.000***	1.000	0.590**	0.467*	0.242		1.000	1.000***
Cluster 7	1.000	1.000	0.849	1.000	0.323	1.000		0.714
Cluster 8	1.000***	1.000**	0.816***	1.000***	0.004	1.000***	0.714	

Significances were obtained by 10,000 permutations and are indicated by asterisks (* < 0.05, ** < 0.01 and *** < 0.001). The *p* values were Bonferroni corrected for multiple comparisons.

The ratio of nonsynonymous to synonymous polymorphisms within *X. mesostena* and within the outgroups was not significantly different from the ratio of nonsynonymous to synonymous polymorphisms fixed between these groups (Fisher's exact test *p* = 1.000). Therefore, results of the McDonald-Kreitman test were consistent with neutral evolution of the *cox1* gene. A highly significantly negative Fu's *Fs* statistic (−35.343, *p*<0.001) indicated population expansion.

### Comparison of phylogeographic patterns found based on AFLP markers and on *cox1* sequences

The five STRUCTURE clusters based on the AFLP data were geographically more distinctly separated than the mitochondrial clades (Fig. 6). This was confirmed by the ANOSIM analyses that showed lower global *R* value for the *cox1* clades than for the ALFP based STRUCTURE clusters and a lack of significant differentiation between some *cox1* clades (Tables 1, 2).

A comparison of the distribution of the AFLP based clusters and the *cox1* clusters showed that the distribution of the clusters southwest of the Psiloritis Mountains, the clusters east of the Dikti Mountains and the more widespread central clusters is somewhat similar. However, a more detailed comparison showed that the boundaries between the clusters are usually not congruent. This means that a haplotype group is not only found in individuals belonging to the AFLP cluster that corresponds geographically to the haplotype clade, but haplotypes of one group can also be found in individuals belonging to adjacent AFLP clusters.

**Figure 7:**
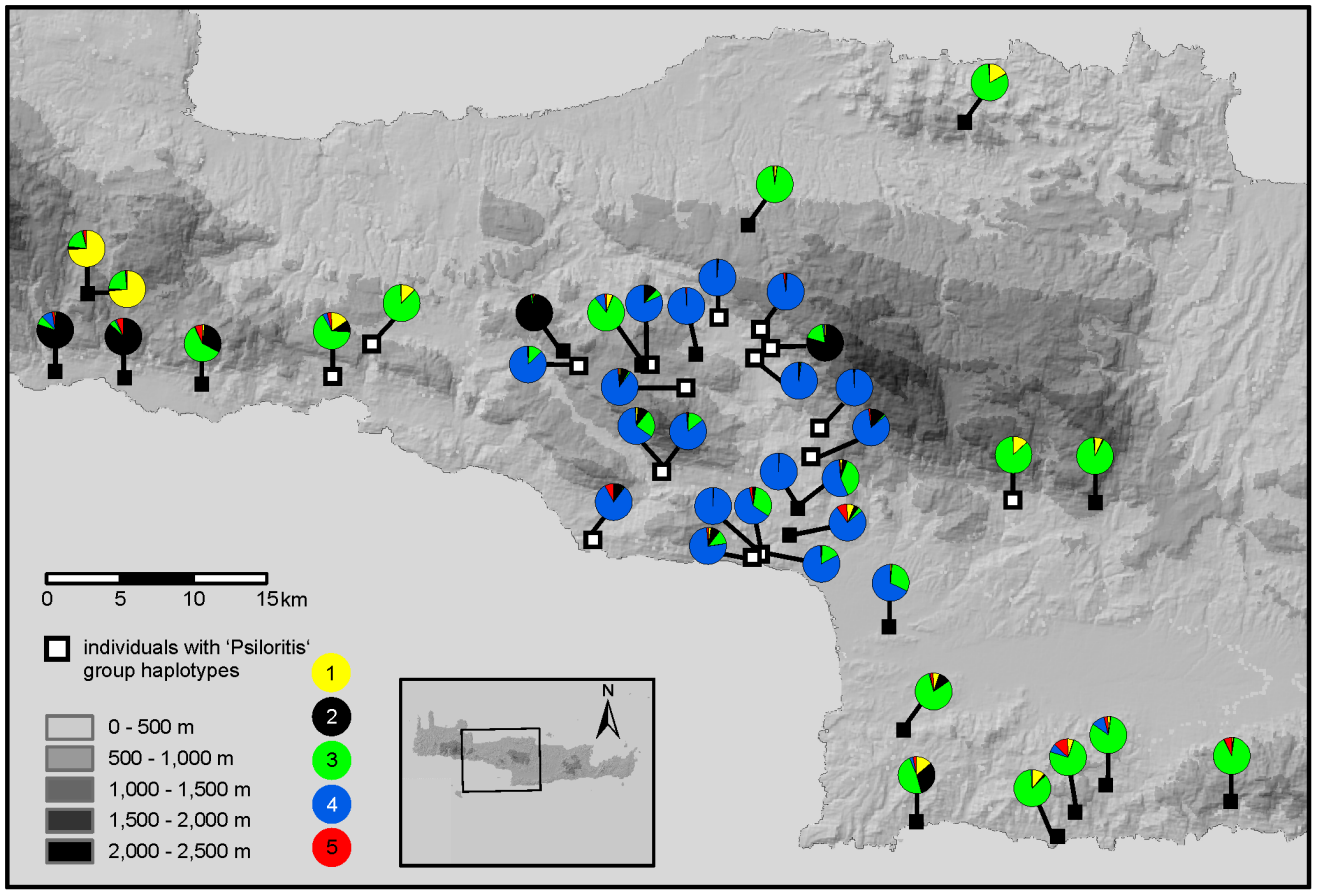
Admixture of the *Xerocrassa mesostena* individuals in the region around the Psiloritis Mountains calculated based on the AFLP data using STRUCTURE. Colours refer to the AFLP based clusters shown in Figures 2A and 4. Individuals possessing ‘Psiloritis group’ *cox1* haplotypes are indicated by white squares, individuals with other haplotypes by black squares.

The situation in the region southwest of the Psiloritis Mountains is particularly complex, because three AFLP clusters meet there (Fig. 7). The ‘Psiloritis group’ *cox1* haplotypes, which are characteristic for this region, were found not only in individuals belonging to the geographically corresponding AFLP cluster, but also in individuals belonging to two additional AFLP clusters (Figs. 3, 5, 7). On the other hand, two other haplotype groups have also been found associated with the AFLP cluster that geographically corresponds to the ‘Psiloritis’ haplotype group.

Moreover, there are some clearly separated haplotype clades that do not correspond with AFLP clusters. For example, the haplotype clade distributed in southern central Crete (Fig. 5B, cluster 6) can be found in individuals belonging to two adjacent AFLP clusters. A lack of congruence between the distributions of AFLP clusters and haplotype clades is also obvious in western Crete. There are two AFLP clusters that are separated by the Lefka Mountains and its foothills and that meet at the west coast of Crete (Fig. 5A, clusters 1 and 2). In contrast, there are three mitochondrial haplotype clades two of which occur both north and south of the Lefka Mountains and its foothills (Fig. 5B, clusters 1–3). The individuals belonging to the southern AFLP cluster are split into a western and an eastern group based on their *cox1* haplotypes. The individuals belonging to the northern AFLP cluster carry even three different *cox1* haplotype clades two of which occur on the Akrotiri peninsula. There are no obvious geographic barriers between the individuals carrying the different haplotype clades.

### Identifying barriers to gene flow and long distance dispersal events

**Figure 8:**
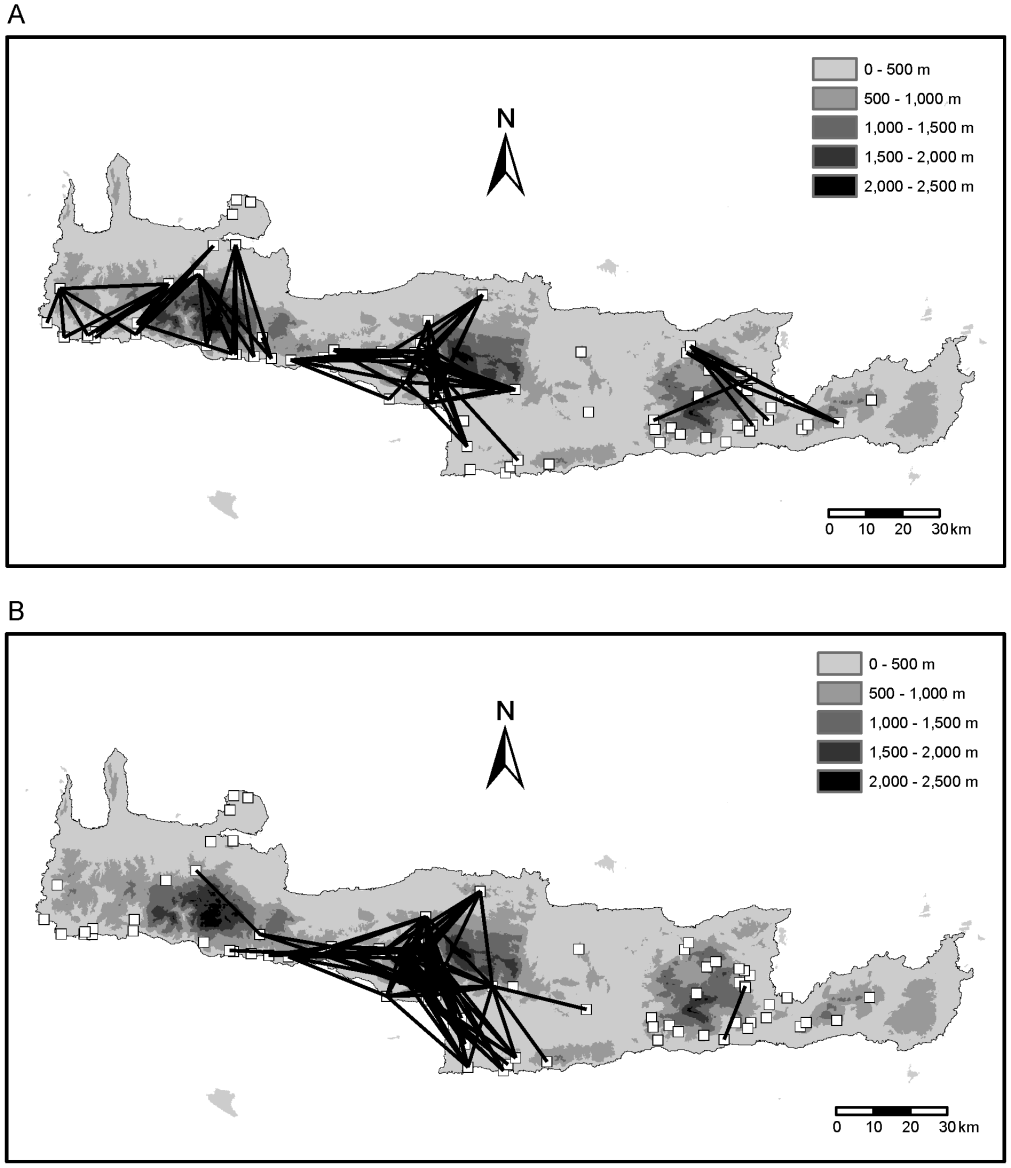
Visualization of geographic barriers by plotting lines between the pairs of individuals that belong to both, the quartile with the highest genetic distances and the quartile with the lowest geographic distances. A. Using Jaccard distances based on AFLP data. B. Using GTR+G distances between *cox1* sequences.

High genetic distances based on AFLP data across short geographical distances that indicate barriers to gene flow are concentrated in three regions of Crete: in the Lefka Mountains, southwest of the Psiloritis Mountains and in the Dikti Mountains (Fig. 8A). With regard to *cox1* sequences, the only region where high genetic distances are concentrated across short geographical distances is the region southwest of the Psiloritis Mountains (Fig. 8B). However, in this region more pairs of individuals show high genetic distances across short geographical distances with regard to the *cox1* sequences than with regard to the AFLP data.

**Figure 9:**
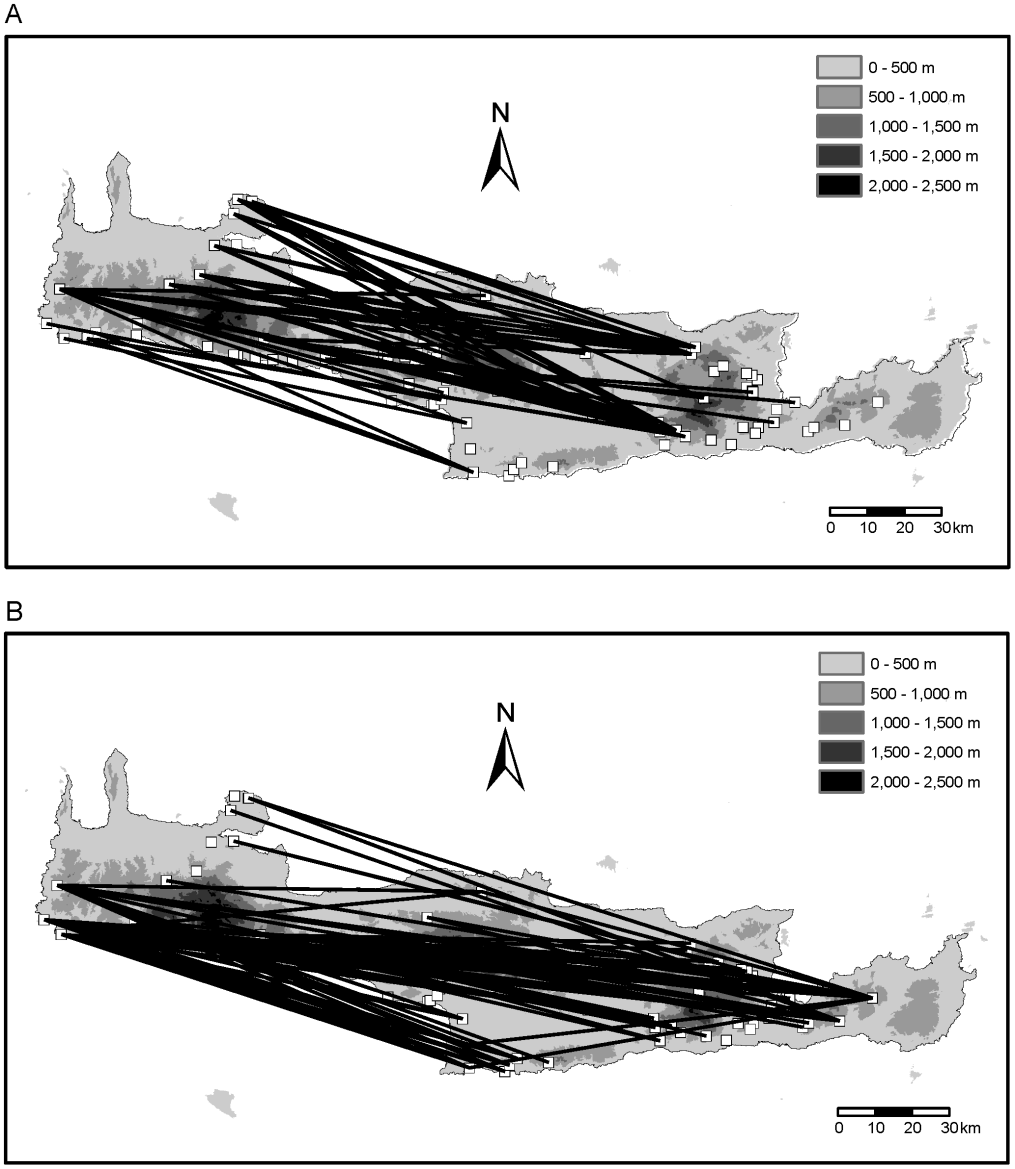
Visualization of potential long distance dispersal events by plotting lines between the pairs of individuals that belong to both, the quartile with the lowest genetic distances and the quartile with the highest geographic distances. A. Using Jaccard distances based on AFLP data. B. Using GTR+G distances between *cox1* sequences.

Low genetic distances based on AFLP data across large geographical distances that indicate potential long distance dispersal events have been found between 63 pairs of individuals. Most of these pairs consist of specimens from western Crete and specimens from the region between the Psiloritis and the Dikti Mountains (Fig. 9A). Many more pairs of individuals (134) show low genetic distances across large geographical distances with regard to *cox1* sequences (Fig. 9B). Most of these pairs of individuals include also one specimen from western Crete. However, the counterparts are not only found in the region between the Psiloritis and the Dikti Mountains, but frequently also in southern central Crete or the region east of the Dikti Mountains.

### Isolation by distance and subdivision into clusters

The distance based redundancy analysis with the genetic distance matrices either based on the AFLP data or the *cox1* sequences as response variables and the geographic coordinates of the sample sites as predictor variables indicated that the proportion of variances of the genetic distances explained by isolation by distance was significant, but low (AFLP: proportion of variation explained was 0.105, pseudo-*F*  =  5.371, *p*<0.001; *cox1*: proportion of variation explained was 0.130, pseudo-*F*  =  6.85, *p*<0.001). A dbRDA with the cluster affiliation of the individuals as predictor variable set and the spatial variables set as covariates showed that the subdivision into clusters explained a higher proportion of genetic variation (AFLP: proportion of variation explained was 0.190, pseudo-*F*  =  5.909, *p*<0.001; *cox1*: proportion of variation explained was 0.560, pseudo-*F*  =  18.96, *p*<0.001). A dbRDA with the genetic distance matrices as response variables and the geographic coordinates of the sample sites as predictor variables restricted to the individual AFLP based clusters or *cox1* haplotype groups showed that isolation by distance explains an additional proportion of the genetic variation within most of the clusters (Tables 3, 4). This proportion was much higher than the proportion of the genetic variation explained by isolation by distance in the complete dataset.

**Table 3:** Results of the dbRDA within the clusters delimited using STRUCTURE based on the AFLP data.

	n	Pseudo-F	*P*	Proportion of variation explained
Cluster 1	11	0.881	0.723	0.181
Cluster 2	17	1.797	0.002	0.204
Cluster 3	24	1.938	0.000	0.156
Cluster 4	20	1.310	0.022	0.134
Cluster 5	23	2.060	0.000	0.171

Significances were obtained by 9,999 permutations.

**Table 4:** Results of the dbRDA within the *cox1* haplotype groups.

	n	Pseudo-F	*P*	Proportion of variation explained
Cluster 1	11	4.181	0.001	0.511
Cluster 2	4	1.165	0.539	0.700
Cluster 3	13	3.305	0.001	0.398
Cluster 4	19	2.351	0.023	0.227
Cluster 5	28	1.934	0.036	0.134
Cluster 6	7	4.075	0.011	0.671
Cluster 8	11	2.373	0.064	0.372

Significances were obtained by 9,999 permutations.

### Association between genetic data and environmental variables

With a significance threshold set to 99% (corresponding to *p*<7.22E-08) MatSAM detected correlations between 4 (0.38%) of the 1307 AFLP loci with at least one of the 105 investigated environmental variables: two with mean monthly percentage of maximum possible sunshine (September and October), one with the coefficient of precipitation of the month August, and one with mean monthly diurnal temperature range of August.

## Discussion

### Mechanisms determining the population structure of land snails

The observed patterns in the population structure of *X. mesostena* can be explained mainly by three mechanisms: population expansion, leptokurtic dispersal and gene flow restriction by geographic barriers. Population expansion (as indicated by the highly significantly negative Fu's *Fs* statistic) was probably facilitated by partial deforestation of Crete in the postglacial. Large parts of Crete were repeatedly forested during more humid periods over the last million years [45]–[47]. Thus, the xerophilous *Xerocrassa* species that occur only in open environments were at least temporarily restricted to woodless patches during these periods. Forest areas may also have formed barriers to gene flow that contributed to the formation of the AFLP clusters and the corresponding *cox1* haplotype clades. Newly available areas were colonized by leptokurtic dispersal [48], i.e. slow active expansion resulting in isolation by distance within the clusters (Tables 3, 4) and occasional long distance dispersal events (Fig. 9) by passive transport [49]. The random long distance dispersal events resulted in departures from the isolation by distance model so that the percentage of genetic variation explained by isolation by distance was low. Gene flow restriction by geographic barriers, in the case of *X. mesostena* the Lefka Mountains, the Psiloriti Mountains, the Dikti Mountains and the Messara plain caused a subdivision of the genepool of *X. mesostena* into five clusters as revealed by STRUCTURE and Gaussian clustering analyses based on the AFLP data (Figs. 1, 2, 4).

Departures from isolation by distance resulting from occasional long distance dispersal have been reported also from other land snail species [50]–[52]. Gene flow restriction by geographic barriers and leptokurtic dispersal [48] are probably major determinants of the population structure of many other wide-spread organisms with low active dispersal capacity, especially soil organisms.

The population structure of *X. mesostena* consisting of many more or less isolated populations that can reach high densities in favourable patches of habitat is typical for many land snail species [8]–[9], [51], [53]. Such population structure is conducive to the persistence of ancestral polymorphisms and, thus, high intraspecific genetic distances as observed in *X. mesostena* [13] because it increases the effective population size [54].

The spatial analysis method implemented in MatSAM [15] did not provide evidence for a strong influence of selection on the population structure of *X. mesostena*. Only 0.38% of the examined AFLP markers show significant correlations with abiotic environmental variables. Two loci are significantly correlated with the percentage of sunshine in relation to maximum day length in September and October. At the end of October, the activity and reproduction period of *X. mesostena* begins after the long dry period of the summer during which the snails aestivate under stones or in bushes. The decreasing percentage of sunshine (or correlated parameters) might act as a trigger for the transition from aestivation to the activity and reproduction period. The results of the spatial analysis method indicate that populations of *X. mesostena* adapted to differences in the onset of favourable conditions for activity and reproduction in different regions of Crete.

The few loci affected by adaptation to environmental parameters cannot explain the geographical subdivision of *X. mesostena*. Another mechanism that might result in geographical differentiation is regionally different selection by predators. Such selection resulted in geographic differentiation of shell shape and sculpture in some land snail taxa [55]–[56]. There is also a large variability in shell shape in *X. mesostena* ranging from high dome-shaped to flat keeled shells [10]. Different shell shapes are often characteristic for populations or groups of neighbouring populations, usually with low variability within populations and sharp differences to other neighbouring groups of populations. However, this variability is observed at a more local scale. There are no consistent shell differences between the major clusters based on the AFLP data [10]. Thus, this clustering cannot be explained by selection by predators. Rather, the geographical boundaries of the AFLP clusters were generally coincident with the most prominent current barriers on Crete, the Lefka, Psiloritis and Dikti Mountains and the Messara Plain (Fig. 5A). This indicates that the strong cluster structure is the result of extrinsically limited gene exchange between the metapopulations forming the AFLP clusters rather than of differential adaptation of these metapopulations.

These results are in agreement with other studies of differentiation processes in land snails indicating that large scale geographical variation in land snails is predominantly caused by geographical separation [3], [7]–[9], [57]. Speciation involving divergent selection and adaptation to different environments seems to be rare in land snails [57]–[59]. In *Cepaea nemoralis* [7], *Partula taeniata* [8], *Theba* on Fuerteventura and Lanzarote [57], and *Charpentieria itala* [9], the barriers that caused the formation of the cluster structure vanished and secondary contact and admixture between formerly separated populations resulted. In contrast, the geographical barriers separating the metapopulation clusters in *X. mesostena* are still limiting gene flow so that the differentiation of separated metapopulations is an ongoing process.

We previously suggested that the Cretan *Xerocrassa* radiation was initiated by sexual selection [11]. Although there are no consistent differences in the genitalia between the *X. mesostena* clusters, the beginning of differentiation can be observed. For example, there are individuals with unusual proportions of the spermatophore forming organs, i.e. a high epiphallus: flagellum ratio (proximal epiphallus: flagellum  =  1.5–2.8), in the cluster occurring east of the Dikti mountains [10]. Such high epiphallus: flagellum ratios have not been observed in any of the other clusters (proximal epiphallus: flagellum  =  0.8–1.6). There are transitions between individuals with unusual high epiphallus: flagellum ratios and individuals with usual epiphallus: flagellum ratios. However, if high epiphallus: flagellum ratios became fixed in this cluster, reproductive incompatibility between this cluster and other clusters and, consequently, differentiation of this cluster into a separate species may result. The population structure of *X. mesostena* suggests that the reduction of gene flow between metapopulations by geographical barriers interacts with sexual selection resulting in the differentiation of these metapopulations into separate species without noticeable ecological differentiation.

There is evidence for gene flow not only between diverging metapopulations within *X. mesostena*, but also at the next stage of differentiation between closely related species. *Xerocrassa franciscoi* and the *Xerocrassa amphiconus/siderensis* group originated recently from *X. mesostena* as indicated by the non-monophyly of their mitochondrial haplotypes with respect to mitochondrial lineages of *X. mesostena* [11]. Nevertheless, the AFLP genotypes of both form clusters separate from that of *X. mesostena*. Both differ from *X. mesostena* mainly in the proportions of the spermatophore forming organs, and they are geographically restricted to small areas at the fringe of the range of *X. mesostena* [10]. A narrow hybrid zone has been observed between the populations of *X. franciscoi* and the neighbouring *X. mesostena* populations [10]. A high percentage of admixture in *X. mesostena* specimens occurring close to the hybrid zone shown by AFLP markers might indicate that there is still some gene flow between the diverging species [13].

The evidence for gene flow between parapatrically distributed Cretan *Xerocrassa* units representing different stages of the speciation process, namely between metapopulations within *X. mesostena* as well as between recently diverged species, indicates that there was always some gene flow during the speciation process. Thus, the ongoing fragmentation of the *X. mesostena* complex might represent an example for parapatric speciation [60]–[61]. The conclusion that evolutionary units can originate or remain distinct despite ongoing gene flow confirms theoretical predictions that speciation is possible in the face of gene flow between differentiating populations [60]–[61]. The lack of ecological differentiation of the Cretan *Xerocrassa* metapopulations and species is also in agreement with the theoretical prediction that divergent selection for local adaptation is not required for rapid speciation [60]–[61], but in the case of *Xerocrassa* sexual selection has probably facilitated the speciation process.

An alternative model would be speciation by vicariance caused by emerging barriers. Under the vicariance model we would expect that other taxa with similar dispersal capacities were affected by the same vicariance events. Thus, the distribution areas of the originating species should be similar and should form distinct geographic clusters. There are radiations of other land snail groups on Crete, e.g., of *Mastus* [62]–[63], *Orculella* [64], and *Albinaria* [65]–[68]. A statistical test indicated that the distribution areas of 74 endemic land snail species belonging to genera with at least two endemic species are not significantly clustered [12]. The lack of geographical concordance between the distribution areas of species belonging to different groups indicates that there is no evidence for speciation by vicariance and, thus, indirectly supports a parapatric speciation model.

### Distribution of mitochondrial haplotype groups and their correspondance with AFLP based clusters

The phylogeographic patterns of the mitochondrial *cox1* gene in *X. mesostena* show some agreement with the pattern found with the AFLP markers, but deviate in other respects. The geographical distribution of some mitochondrial haplotype groups agrees closely with that of some AFLP clusters (Fig. 6). Slight differences between the distribution of mitochondrial haplotype groups and the corresponding AFLP clusters as found, e.g., in the region southwest of the Psiloritis Mountains and east of the Dikti Mountains (Fig. 5, mitochondrial clusters 4 and 8, AFLP clusters 4 and 5) can be explained by limited dispersal across a barrier or dispersal after the erosion of a barrier. Similar patterns have also been found in other studies comparing the distribution of nuclear and mitochondrial markers across boundaries of phylogeographic groups [69]–[71]. This also demonstrates that the mitochondrial haplotype groups of *X. mesostena* do not represent distinct cryptic species despite the large genetic distances separating them.

Each migration event may result in the establishment of the mitochondrial lineage in the receiving population. In contrast, migrating individuals are not likely to cause a noticeable lasting effect on the multilocus pattern in the receiving population, even if this immigrant propagates successfully because its multilocus genotype will be erased by recombination so that after a few generations hardly any traces of the dispersal may be detectable. The much higher number of potential long distance dispersal events suggested by the mitochondrial data (Fig. 9B) vs. the multilocus data (Fig. 9A) for the same individuals underscores the different effects of migration on single gene patterns and multilocus patterns. Within clusters, where dispersal is more frequent, gene flow will result in isolation by distance as we have found with both the AFLP data and the mitochondrial sequences (Tables 3, 4).

Some phylogeographic breaks in the distribution of the mitochondrial haplotype groups of *X. mesostena* do not correspond with the pattern shown by the multilocus data (Fig. 5) and cannot be explained by dispersal of a few individuals across a barrier. For example, the distribution of the haplotype group of *X. mesostena* in southern central Crete (Fig. 5B, cluster 6) does not have a multilocus equivalent, but runs across the boundaries of the AFLP based clusters. Several processes may result in phylogeographic breaks in a single locus marker that do not correspond with breaks in a multilocus dataset: (1) selection on the locus [72]–[73]; (2) introgression [74]–[79]; (3) gene flow restriction by barriers that are so young that most other markers have not yet been sorted (which may become more apparent when a marker with a lower effective population size like mitochondrial DNA is studied); (4) geographical differentiation of mitochondrial DNA in sedentary females combined with homogenization at nuclear loci by dispersal of males [80]; (5) random subdivision [81]–[83].

There is no evidence that the distribution of the haplotype group of *X. mesostena* in southern central Crete (Fig. 5B, cluster 6) is the result of selection, because McDonald-Kreitman test results were consistent with neutral evolution of the *cox1* sequences. However, we cannot exclude that there was selection on other linked mitochondrial loci. It cannot be ascribed to introgression because this haplotype group is not shared with other *Xerocrassa* species [11]. The distribution of the haplotype group of *X. mesostena* in southern central Crete can also not be ascribed to a recent barrier to gene flow. Rather, the distribution of this haplotype group extends across a current barrier, the Messara Plain, the southern border for the ‘Psiloritis’ haplotype group (Fig. 5B, cluster 4) and the corresponding AFLP based cluster (Fig. 5A, cluster 4). The lack of a multilocus based cluster corresponding to the *cox1* haplotype group cannot be explained by a homogenization of the nuclear markers as a result of male dispersal, because *X. mesostena* is a hermaphrodite in which each individual contributes to the transmission of mitochondria. Thus, it seems that the boundaries of the haplotype group of *X. mesostena* in southern central Crete represent a random phylogeographic break. Computer simulations have shown that such random phylogenetic breaks in the distribution of single locus markers have to be expected and that they originate especially easily in species with low dispersal abilities like land snails [53], [84] even when they are continuously distributed and even when there are no barriers to gene flow [81]–[83]. Nevertheless, phylogenetic breaks in the distribution of single locus markers that do not correspond with current barriers to gene flow have been ascribed with few exceptions (e.g., [85]) to past barriers to gene flow. If there were past barriers to gene flow, other markers should also show a phylogeographic break in that region. This is not the case for the haplotype group of *X. mesostena* in southern central Crete as we could show with the help of the AFLP data.

The frequent cases in which mitochondrial haplotype groups were found in different nuclear backgrounds in *X. mesostena* support the problem suggested in several studies arguing against using a single genetic marker for reconstructing the phylogeographic history of species [77], [86]–[90]. Decisions, e.g. concerning conservation issues like breeding programs or re-introductions, should not be based on phylogeographic patterns observed only with a single genetic marker. Obviously, the strongest possible conclusion, namely that two groups characterized by different haplotype clades represent separate species should also be based on multilocus data. Molecular taxonomy [91]–[92] based on mitochondrial DNA alone would have resulted in a splitting of *Xerocrassa mesostena* into several ‘species’ [93] that are neither isolated from each other nor reflect the phylogeographic structure revealed by multilocus data.

## Supporting Information

Table S1 Locality, voucher, *cox1* data and AFLP data for the used *Xerocrassa* individuals (xls).
(XLS)

Table S2 Environmental parameters and AFLP data used for the MatSAM analysis (xls).
(XLS)
